# XMRV: usage of receptors and potential co-receptors

**DOI:** 10.1186/1742-4690-8-S1-A224

**Published:** 2011-06-06

**Authors:** Mohan K H G Setty, Krishnakumar Devadas, Ragupathy Viswanath, Veeraswamy Ravichandran, Shixing Tang, Owen Wood, Durga S Gaddam, Sherwin Lee, Indira K Hewlett

**Affiliations:** 1 Center for Biologics Evaluation and Research, Food and Drug Administration, Bethesda, MD, 20892, USA

## Background

XMRV is a gammaretrovirus first identified in prostate tissues of prostate cancer(PC) patients and later in the blood cells of patients with Chronic Fatigue Syndrome (CFS). Since its discovery there have been controversial reports of detection in both PC and CFS. Although XMRV is thought to use XPR1 for cell entry, it infects A549 cells that do not express XPR1, suggesting usage of other receptors or co-receptors. The aim of the study was to determine receptor and co-receptor usage to elucidate the mode of entry, transmission, infectivity of XMRV in different cell types and to determine cellular tropism.

## Methods

To study the usage of different receptors and co- receptors that could play a role in XMRV infection of lymphoid cells and GHOST (GFP- Human osteosarcoma) cells expressing CD4 along with different chemokine receptors including CCR1, CCR2, etc., were infected with XMRV. Culture supernatants and cells were tested for XMRV replication using real time quantitative PCR.

## Results

Infection and replication of XMRV was seen in a variety of GHOST cells, LNCaP, DU145, A549 and Caski cell lines. The levels of XMRV replication varied in different cell lines showing differential replication in different cell lines. However, replication in A549 which lacks XPR1 expression was relatively high. XMRV replication varied in GHOST cell lines expressing CD4 and each of the co- receptors CCR1 – CCR8 and Bob.

## Conclusion

XMRV replication was observed in GHOST cells that express CD4, and each of the chemokine receptors ranging from CCR1- CCR8 and Bob suggesting that infectivity in hematopoietic cells could be mediated by use of these receptors. Infection of Lung epithelial cell A549 lacking XPR1 expression clearly indicates usage of other receptors by XMRV for entry into susceptible cells.

